# Are PARPs promiscuous?

**DOI:** 10.1042/BSR20212489

**Published:** 2022-05-06

**Authors:** Karla L.H. Feijs, Roko Žaja

**Affiliations:** Institute of Biochemistry and Molecular Biology, RWTH Aachen University, Pauwelsstrasse 30 Aachen, Germany

**Keywords:** ADP-ribosylation, amino-acid specificity, hydrolase, PARPs

## Abstract

Post-translational modifications exist in different varieties to regulate diverse characteristics of their substrates, ultimately leading to maintenance of cell health. The enzymes of the intracellular poly(ADP-ribose) polymerase (PARP) family can transfer either a single ADP-ribose to targets, in a reaction called mono(ADP-ribosyl)ation or MARylation, or multiple to form chains of poly(ADP-ribose) or PAR. Traditionally thought to be attached to arginine or glutamate, recent data have added serine, tyrosine, histidine and others to the list of potential ADP-ribose acceptor amino acids. PARylation by PARP1 has been relatively well studied, whereas less is known about the other family members such as PARP7 and PARP10. ADP-ribosylation on arginine and serine is reversed by ARH1 and ARH3 respectively, whereas macrodomain-containing MACROD1, MACROD2 and TARG1 reverse modification of acidic residues. For the other amino acids, no hydrolases have been identified to date. For many PARPs, it is not clear yet what their endogenous targets are. Better understanding of their biochemical reactions is required to be able to determine their biological functions in future studies. In this review, we discuss the current knowledge of PARP specificity *in vitro* and in cells, as well as provide an outlook for future research.

## ADP-ribosylation as a post-translational modification

Cells respond to signals from both within as well as from the outside to maintain their fitness. This includes reacting to potential stressors such as viruses, lack of nutrients or DNA damage, as well as for example growth stimulating factors. One way to react to such stimuli is to modify existing proteins with small chemical groups such as phosphate in phosphorylation, or with small proteins such as ubiquitin. These modifiers can alter any protein characteristic: localization, degradation, activity and interactors are some examples of the effects post-translational modifications can have to regulate proteins and thereby cell health. ADP-ribosylation is a modification which exists in two flavors: proteins can be modified with either one ADP-ribose (ADPr) moiety, referred to as mono(ADP-ribosyl)ation or MARylation, or with a chain of ADPr, termed poly(ADP-ribosyl)ation or PARylation [1]. In cells, this reaction is performed by ADP-ribosyltransferases (ARTs) of the poly(ADP-ribose) polymerase (PARP) enzyme family. This name once was an abbreviation for poly(ADP-ribose) polymerase and has been given to this protein family at a time when all enzymes were thought to be able to modify targets with PAR [2,3]. Later on, this turned out to be incorrect as it became clear that the majority of enzymes in this family transfer only one ADPr to their targets [4–6]. Recent studies have indicated that not only proteins, but also nucleic acids can serve as acceptor for ADPr as summarized elsewhere [7,8]. It is not clear whether this modification of nucleic acids occurs in cells and as such the biological function of nucleic acid MARylation remains to be identified.

**Figure 1:**
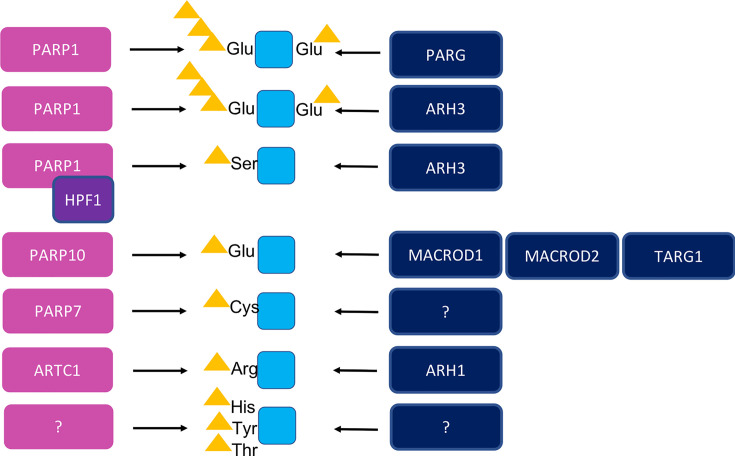
Schematic overview of the specificity of the ADP-ribosylation reaction performed by ADP-ribosyltransferases and hydrolases PARP1 generates chains of poly(ADP-ribose) on glutamates, association with HPF1 changes its specificity to modification of serine. PARG can degrade PAR chains but not remove the protein-linked ADP-ribose. ARH3 reverses both PARylation as well as removes ADP-ribose from serine. PARP10 modifies acidic residues and is counteracted by macrodomain-containing hydrolases MACROD1, MACROD2 and TARG1. PARP7 modifies cysteines with unidentified counteracting enzyme. The extracellular ARTC1 modifies arginines which the intracellular ARH1 can reverse. It is not known whether additional intracellular enzymes modifying arginine exist, or whether ARTC1 substrates can become cytosolic to be demodified by ARH1. Histidine, tyrosine and threonine modifications have been identified using mass spectrometry; however, the respective transferases and hydrolases have not yet been identified.

The modification of proteins as well as nucleic acids with ADPr or polymers thereof is reversible, with two distinct families of enzymes performing this reaction in cells [9]. The enzymes MACROD1, MACROD2 and TARG1 contain a so-called macrodomain and were described to reverse the attachment of ADPr to acidic residues in a hydrolytic reaction [10–13], whereas the other macrodomain-containing enzyme PARG cleaves the glycosidic bond between ADPr moieties and thereby degrades PAR chains [9] (Figure 1). The structurally unrelated ADP-ribosylhydrolase (ARH) family contains three members [14,15]. ARH1 reverses the modification from arginine [16,17], ARH3 can reverse both serine modification [18] as well as degrade PAR chains and for ARH2 no activity has been documented yet [14]. The source of intracellular arginine modification is not known yet, as all ARTs known to date that modify arginine, the ARTCs, reside at the extracellular membrane [19,20]. ADP-ribosylation was first identified in the 1960s [21] and already in the 1980s two different types of ADP-ribosylation were already discussed [22]; however, the predominant modification in cells was long thought to be modification of acidic residues. We now know however that PARP1 when complemented with co-factor HPF1 modifies serine [23] and that for example PARP7 is preferentially modifying cysteine residues [24,25] (Figure 1). Recent reviews have focused on the biological function of both the poly- and mono-ARTs [1,26,27], on the hydrolase families [9] and on the potential roles of both hydrolases and transferases in diverse diseases including cancer [16,28–30]. Despite recent efforts, it is not entirely clear which amino acids are modified with ADPr by which enzymes. Mass spectrometry studies have identified a large number of proteins, which are modified on a range of amino acids, including basic, acidic as well as uncharged amino acids as acceptor [18,24,31–36]. In this review, we intend to give an up-to-date overview of the substrate specificity of the PARPs, as well as discuss future challenges that need to be addressed to increase our understanding of the modification ADP-ribosylation.

## PARP1/HPF1 toward serine

PARP1 is the enzyme of the PARP family that has been best studied. Initially thought to be a polymerase generating chains of ADPR, later on this activity was determined to be an iterative transferase activity instead [3,6,37]. PARP2 can take over many of PARP1’s functions in cells, as underscored by the fact that PARP1 or PARP2 knockout mice are viable, but double knockout mice are not [38]. PARP1 is a key enzyme in DNA damage repair, where it generates local PAR-chains which in turn attract key players required to resolve DNA damage, such as BRCA1 [39,40]. PARP1/2 inhibitors are at present being used in the clinic to treat cancer patients with BRCA mutations [41], as additional loss of PARP1 disables functional DNA repair in the cancer cells specifically. Also PARG, the enzyme reversing PARylation in cells, is under investigation as cancer drug target [28]. The latest discovery concerning PARP1, is the fact that when partnered with HPF1, it becomes an enzyme which modifies proteins on serine residues instead of the acidic residues it was described to modify [23]. Structures of PARP1 or PARP2 in complex with HPF1 showed that the presence of HPF1 blocks chain formation by sterical hindrance [42]. If HPF1 is present in excess in *in vitro* experiments, it can even turn PARP1 into an NAD^+^ hydrolase [43], with hitherto unknown occurrence and relevance in cells. The biological function of serine ADP-ribosylation has not been addressed in detail yet; however, it may be part of the histone code, due to the fact that MARylated serines are potential phosphate acceptors [44], as well as the often found “KS” motif where the lysine can be an acceptor for different modifications such as acetylation and methylation [45]. Future studies are required to study these possibilities in detail. The fact that HPF1 can alter PARP1’s substrate specificity so dramatically also carries consequences for the other PARP family members, as a similar principle may apply to them: binding of a co-factor may complement their catalytic site and thereby alter their substrate-specificity. The known information about PARP1, HPF1 and the DNA damage response was recently summarized [46]. The specificity of PARP1 has thus been expanded from exclusively PAR on glutamates to include MAR on serine residues. Less certain is the target residue of the reaction catalysed by the mono-ARTs.

## PARP10

**Figure 2:**
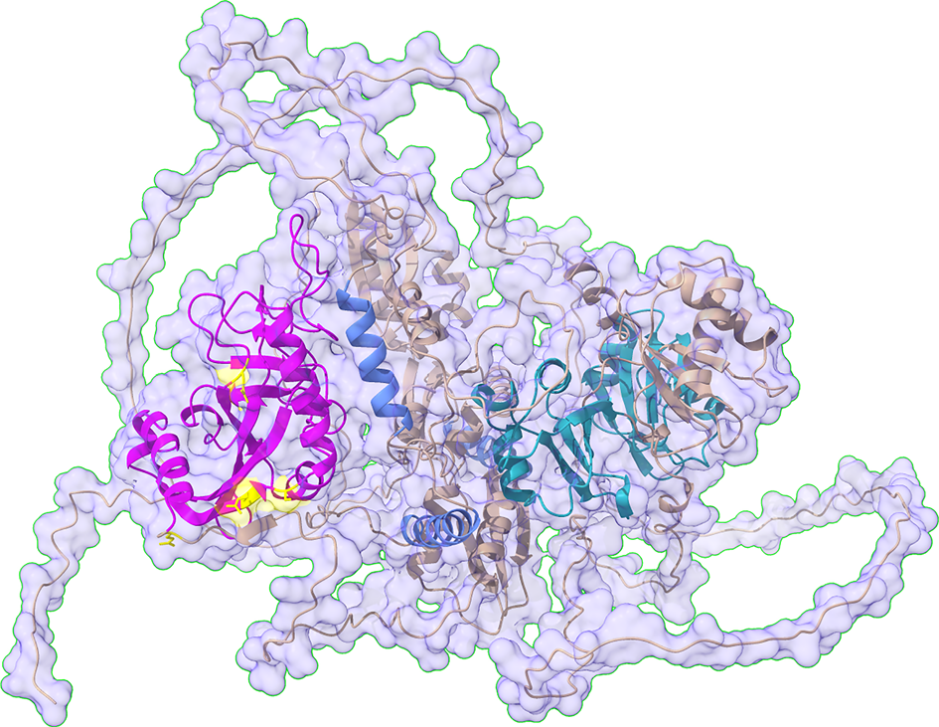
The predicted structure of PARP10 with highlighted globular domains The structure of PARP10 predicted by Alphafold was downloaded [66] and edited using ChimeraX [67], to annotate the predicted and known domains and modification sites. Domains were analyzed using Pfam/SMART [68]. The catalytic domain is highlighted in magenta, the ubiquitin interaction motifs (UIM) in skyblue and the RNA recognition motifs (RRM) in turquoise. Mapped automodification sites are all in the catalytic domain and are highlighted in yellow. The surface of the molecule is rendered 80% transparent to be able to appreciate the bulkiness of the molecule and at the same time visualize the domain architecture. The prediction of the flexible regions is highly uncertain. The structure was downloaded from Alphafold on 17.11.2021; the Pfam predictions on 17.11.2021.

The first PARP to be characterized as mono-ART, was PARP10 [4]. PARP10 mRNA expression is up-regulated by interferon α [47,48]. The protein was described to inhibit NF-kB signaling [49] and to repress replication of certain single-stranded RNA viruses including chikungunya virus [47,50,51]. PARP10 has also been studied in the context of DNA damage, as it interacts with PCNA and is required for translesion DNA synthesis [52]. Loss of PARP10 leads to genomic instability and enhanced sensitivity to DNA damaging agents [52]. These findings were corroborated by the hypersensitivity to DNA damage of cells from a patient lacking functional PARP10 [53]. Finally, overexpression of PARP10 leads to apoptosis in HeLa cells [54], whereas it promotes cell proliferation in RPE-1 cells [55]. Its molecular function is thus not entirely clear and may depend on cell-specific properties. To determine PARP10 specificity toward different amino acids, the catalytic domain of PARP10 was subjected to an automodification reaction and subsequently exposed to different chemicals including neutral hydroxylamine, which is known to release the modification from acidic residues [4]. It was concluded that PARP10 modifies glutamates or aspartates. It needs to be noted that even after 4 h of exposure to neutral hydroxylamine, a residual signal is still present. The halftime of the MARylation on glutamates is around 10 min in neutral hydroxylamine and with longer incubation times also arginine modification will be lost [56–58]. This experiment can thus not differentiate between glutamate and arginine modification, especially considering that the shortest time-point analysed here was 1 h [4]. In a later work focusing on hydrolases, MACROD2 was shown to reverse the automodification signal on PARP10 but also not fully [12]. If indeed the macrodomain-containing hydrolases MACROD1, MACROD2 and TARG1 reverse modification of acidic residues, the question remains what the residual signal may be. In a mass-spectrometry based analysis of PARP10 automodification, glutamate, arginine and serines were identified as modification sites [59], which forms a putative explanation for the remaining signal after hydrolase incubation. However, sequential incubation of PARP10 with first MACROD1 and then ARH1, ARH3, PARG or combinations thereof did not lead to a further reduction of the signal [60]. Non-enzymatic modification can occur, especially on lysine [61], which could account for the hydrolase-resistant signal. The predicted structure of PARP10 (Figure 2) highlights the complexity of these enzymes, wherein activity may well be regulated by different arrangements of the diverse domains and the large disordered regions, depending on for example posttranslational modifications or co-factors. Despite considerable effort of diverse labs in the field, it thus still remains unclear what the target of PARP10 may be: can it truly modify diverse amino acids, perhaps dependent on specific co-factors, or is this promiscuity a result of *in vitro* and overexpression artifacts? One aspect that may cloud these findings, is the fact that most studies thus far rely on a truncated version of the enzyme purified from *Escherichia coli*, which may introduce artifacts not seen with the full-length protein in its natural environment. Considering the bulkiness of the whole protein compared to the isolated catalytic domain, colored magenta in Figure 2, it is perhaps not surprising that some specificity is lost when only the catalytic domain is used for *in vitro* assays. Several studies addressed the biological function of PARP10 by investigating its substrates [62]: it was reported to MARylate and thereby inhibit GSK3β [12,63], although at the time no reliable mass spectrometry methods nor detection reagents existed to confirm modification of GSK3β in cells, may likewise regulate Aurora A kinase activity [63,64], it may have a role in immunity [47–49], and it was reported to be involved in DNA replication and repair [52,53,55]. In none of these works modification sites of targets were identified, which will be a challenge for future studies intending to further define PARP10 function. The other open question is how specificity is achieved: from the *in vitro* assays, it appears that PARP10 is quite promiscuous, modifying for example a range of growth factors and other proteins which it is unlikely to ever encounter in cells due to their different intracellular localization [63], as well as automodify on a range of different amino acids [59,60]. Specificity may be achieved by quick reversal by hydrolases, leaving only certain proteins ADP-ribosylated, or it could be regulated by highly restricted access to targets. An example hereof is formed by the ARTCs, which are present in lipid-rafts and have access to various receptors present there, but not to for example cytoplasmic proteins [65]. Alternatively, specificity may be conferred by a co-factor similar to the altered activity of PARP1 when HPF1 is bound [42]. Future studies will have to discern between these possibilities, however in meantime *in vitro* assays with recombinant proteins need to be interpreted with caution.

## PARP7

PARP7 was initially described as gene up-regulated by the environmental toxin 2,3,7,8-tetrachlorodibenzo-*p*-dioxin (TCDD) [69], whose encoded protein can serve as co-activator of liver X receptor (LXR) [70] and as repressor of aryl hydrocarbon receptor (AHR) and of the estrogen receptor alpha [71–73]. PARP7 activity is stimulated by TCDD and in turn leads to repression of AHR function [74]. Confirming a key role for PARP7 in the AHR-mediated response to dioxins is the fact liver-specific PARP7 knockout mice are more sensitive to dioxins [75]. Although traditionally studied in the response to dioxin exposure, recent research has expanded on different roles for AHR including in immunity [76]. PARP7 is one of the interferon-induced PARPs, which hints at a role for PARP7 in immunity [47]. In a colitis mouse model, Parp7^−/−^ mice displayed a milder phenotype, possibly by recruiting immune cells to the site of inflammation [77]. PARP7 was furthermore reported to MARylate TBK1, a major kinase activated during the type I interferon response, leading to inhibition of TBK1 and thereby suppressed interferon response [78]. Inhibition of PARP7 using a small molecule inhibitor in a lung cancer xenograft model led to complete regression due to activated interferon signaling [79]. PARP7 is thus involved in the cellular response to TCDD exposure as well as in immunity and may be targeted by small molecules as anticancer treatment. PARP7 substrates were identified using mass spectrometry in two studies to define the molecular pathways through which it functions [24,80]. One was performed in ovarian cancer cells, where it was reported to MARylate α-tubulin to regulate tubulin stability [80], whereas in the other study in HEK293 cells many substrates with a role in immunity were identified [24]. In the latter work the modified proteins were further analysed to determine the exact modification sites, where the majority of sites mapped are cysteines. The identification of cysteine as major acceptor of PARP7-mediated MARylation agrees with an earlier study, which identified cysteine 39 as one of the PARP7 automodification sites [25]. The cysteine MARylation introduced by PARP7 appeared more stable in cells than the modification of acidic residues, prompting the authors to speculate that the amino acid modified may influence the duration of the modification [24]. It must be noted that for cysteine MARylation no hydrolase is known. Either this enzyme has not been identified yet, or the lack of a cysteine-ADPr reversing enzyme might explain why this modification appears more stable and more abundant. The here summarized studies of PARP10 and PARP7 underscore the potential functions they have in diverse processes such as antiviral immunity, dioxin toxicity and DNA damage response; however, they also convey how little we still know about the mechanisms through which the majority of intracellular mono-ARTs function.

## Chemical genetics and mass-spectrometry based studies to identify endogenous ADP-ribosylated proteins

As briefly mentioned above, it was thought previously that PARPs modify acidic or basic residues [2,4,56,81–83], which has been challenged by different specificities of PARP1/HPF1 [23], PARP7 [24,25,80] and PARP10 [59,60]. Investigating all PARP enzymes using traditional biochemical methods such as chemical and hydrolase treatments may be highly time-consuming and error-prone, as it again relies on high amounts of recombinant enzyme analysed in artificial settings. The solution may be the implementation of in-cell investigations based on either chemical genetics or by mass-spectrometry based analysis. For chemical genetics, the catalytic sites of certain ARTs have been designed to utilize only a specific NAD^+^-derivative, which will lead to the confident identification of substrates of this ART only [84]. These chemical engineering tools were used to identify substrates of DNA-damage associated PARPs such as PARP1-3 and have also been used to map in-cell substrates of the mono-ARTs PARP7, PARP10, PARP11 and PARP14 [24,80,84–86]. There was no substantial overlap between targets of different PARPs, indicating that they are active in different cellular processes. These approaches are very useful to identify specific substrates of specific ARTs while excluding the possibility that overexpression of one PARP leads to activation of another, however a drawback of these studies is that the cells need to be permeabilized to allow the NAD^+^ analogues to enter the cells, as well as overexpress a mutant enzyme. The alternative is to measure endogenous ADP-ribosylation, for which several mass spectrometry approaches have been used. ADP-ribose is prone to fragmentation when analysed using standard collision-induced dissociation (CID) mass spectrometry [87]. Better results have been obtained using electron-transfer dissociation (ETD), high-energy collisional dissociation (HCD), electron-transfer higher-energy collisional dissociation (EthcD) or the latest approach using activated ion electron transfer dissociation (Ai-ETD) [33], although serine modification is labile using HCD [88]. Early mass spectrometry-based studies identified mainly acidic residues, partially due to the particular enrichment methods used, which were biased toward acidic residues [89–91]. The presumption that ADP-ribosylation occurs on acidic or basic residues also clouded data analysis, as the standard software will only assign the modification to residues it is programmed to look for. These approaches can be optimized by nonlocalized searching of the data, which was suggested to both enhance the identification of ADP-ribosylated peptides, as well as avoid mislocalization [31]. Besides different fragmentation and analysis techniques, also sample preparation has been divergent. A major breakthrough was the usage of the macrodomain-containing protein Af1521 from *Archaeoglobus fulgidus* to enrich modified proteins from mammalian cells before analysis [92], which has been further optimized [93]. Using this enrichment method and different dissociation methods including ETD and EThcD, over 7000 modification sited were mapped in more than 11,000 unique proteins in HeLa cell lysates, implying that the ARTs are promiscuous in cells as well [34]. Modified amino acids identified are mostly serine, but also basic amino acids arginine and lysine, acidic amino acids glutamate and aspartate, as well as cysteine, tyrosine, histidine and threonine. When analysing HPF1 knockout cells, the proportion of serine modification drops from 86% to 50%, implying that although HPF1 is a major contributor to serine modification, it still occurs without it. In ARH3 knockout cells, serine modification goes up to as much as 98% of all modification measured, indicating that this is the major eraser [32]. Mass spectrometry-based identification of ADP-ribosylated proteins and the exact modification site therein, has thus improved tremendously over the last decade. It appears that identifying modified proteins is not a problem anymore; however, the question now becomes what the functional consequence is of modification for the identified proteins. Of the thousands of identified ADP-ribosylated proteins, only very few have been characterized in more detail to determine how the modification affects the modified protein. Careful analysis is also required to determine whether the chosen enrichment and mass spectrometry methods will confer a bias towards detecting specific amino acids, either through a higher affinity of the enrichment module toward some modified amino acids over others, or via the fragmentation and analysis methods applied.

## Outstanding questions and future outlook

**Figure 3:**
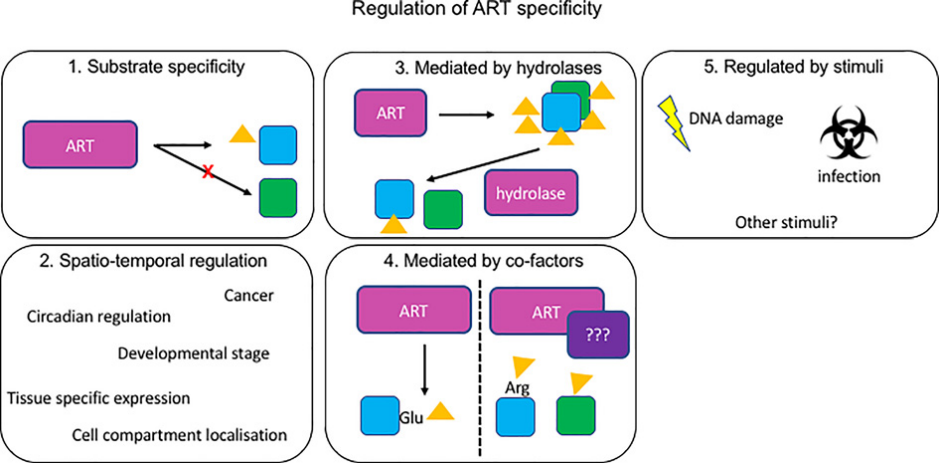
Possible ways of regulating ART activity 1. Intracellular ARTs are highly specific and modify only certain substrates. 2. ARTs are promiscuous but their activity is restricted by restricted expression during specific developmental stages, tissues and diseases or by localization to specific compartments. 3. ARTs are promiscuous and selectivity is achieved by specific reversal by hydrolases. 4. Binding of co-factors alters substrate specificity. 5. ARTs are activated toward specific targets by certain stresses such as DNA damage and infection.

Possibly the largest challenge is thus the confident annotation of MARylation on specific amino acids of specific targets by the different PARP enzymes and the elucidation of the consequence thereof for individual molecules. The solution here is most likely not the mass spectrometric analysis of individual, recombinant enzymes with recombinant substrate proteins incubated with NAD^+^ *in vitro* due to aforementioned potential artifacts, but rather the analysis of the modification present in cells. For the majority of mono-ARTs it is not clear which amino acids they modify and for most studied to date, more than one acceptor amino acid was reported to be modified. There are several putative explanations for this: (a) these enzymes are promiscuous, (b) they are specific but their association with co-factors similar to HPF1 shifts their specificity or (c) the measured diversity of amino acids modified is an artifact produced by the employed experimental techniques. Current mass spectrometry approaches need to be combined with for example the described chemical genetics strategies to be able to attribute modifications measured to specific enzymes, as has been done for some PARPs [84]. This will probably have to be supplemented by hydrolase knockdown or inhibition, as fast reversal of the modification by these enzymes may lead to false negative data, as does PARP activity during lysis [60]. Once additional stimuli have been identified which lead to endogenous ART activation, mass spectrometry using cells with engineered ARTs may help identifying the relevant, specific ART substrates and modification sites. Ideally, cells where individual enzymes have been removed will be analyzed, to not introduce artifacts by transferase overexpression and still gain information on the contribution of individual enzymes to the overall ADP-ribosylome. One intriguing possibility is that the ARTs are indeed highly promiscuous, modifying any suitable residue that crosses their path especially upon high overexpression, and that specificity is regulated spatially as well as by the counteracting hydrolases (Figure 3). This might also explain why ADP-ribosylhydrolases are found in distinct cellular compartments such as cytosol, nucleoli and mitochondria [94]. Mass spectrometric analyses of cells lacking or overexpressing combinations of specific transferases and hydrolases will be essential to clarify this possibility.

With confident identification of targets modified by specific enzymes in response to certain stimuli, the next step will be to elucidate the consequence of the modification for individual proteins. Does the modification of specific amino acids always lead to the same effect, such as inactivation, relocalization or degradation of the modified protein? Or does every modification lead to a different effect depending on the target and amino acid modified? As it stands, few substrates have been characterized in detail, leaving the significance of the modification ADP-ribosylation for individual targets and their importance for cell health unclear. To fully understand ADP-ribosylation, we need to clarify the biochemical mechanism and specificity of individual ARTs (Figure 3) as well as start investigating the consequence of modification for individual target molecules. Future work will thus have to demonstrate whether the endogenous ARTs are as promiscuous in cells as they are in *in vitro* assays.

## Abbreviations

ADPr: ADP-ribose
AHR: aryl hydrocarbon receptor
Ai-ETD: activated ion electron transfer dissociation
CID: collision-induced dissociation
ETD: electron-transfer dissociation
EthcD: electron-transfer higher-energy collisional dissociation
HCD: high-energy collisional dissociation
LXR: liver X receptor
PARP: poly(ADP-ribose) polymerase
TCDD: 2,3,7,8-tetrachlorodibenzo-*p*-dioxin
